# Current Challenge: Endoscopic Submucosal Dissection of Superficial Non-ampullary Duodenal Epithelial Tumors

**DOI:** 10.1007/s11864-020-00796-y

**Published:** 2020-10-26

**Authors:** Kazuya Akahoshi, Masaru Kubokawa, Kazuki Inamura, Kazuaki Akahoshi, Yuki Shiratsuchi, Shinichi Tamura

**Affiliations:** 1 Endoscopy Center, Aso Iizuka Hospital, 3-83 Yoshio town, Iizuka, 820-8505 Japan; 2 Department of Gastroenterology, Aso Iizuka Hospital, Iizuka, 820-8505 Japan; 3 Faculty of Medical Engineering, Aso Iizuka Hospital, Iizuka, 820-8505 Japan

**Keywords:** Early duodenal cancer, Duodenal adenoma, Endoscopic submucosal dissection, Endoscopic mucosal resection, Clutch cutter, Superficial non-ampullary duodenal epithelial tumor

## Abstract

With the widespread use of esophagogastroduodenoscopy in recent years, the detection rate of superficial non-ampullary duodenal epithelial tumors (SNADETs) is increasing. Most SNADETs are early-stage adenocarcinoma or benign conditions, including adenoma. Therefore, endoscopic resection is desirable from the perspective of quality of life. However, endoscopic resection for SNADETs has not yet been established. Endoscopic submucosal dissection (ESD) is the most promising method for the treatment of SNADETs, because ESD provides a high rate of en bloc resection and a low rate of recurrence regardless of the tumor size. However, the difficulty of the procedure and a high rate of severe adverse events including perforation and bleeding have become major problems. Various preventive countermeasures for adverse events, such as use of specific devices, endoscope stabilization methods, and endoscopic closure of the post-ESD ulcer using clips, are reported to reduce the risk of the adverse events of ESD for SNADETs. This article reviews and highlights the current state of ESD for SNADETs and new challenges towards safe and effective ESD.

**Electronic supplementary material:**

The online version of this article (10.1007/s11864-020-00796-y) contains supplementary material, which is available to authorized users.

## Introduction

Widespread use of esophagogastroduodenoscopy (EGD) has resulted in increased detection of superficial non-ampullary duodenal epithelial tumors (SNADETs) [1•, 2, 3]. SNADETs include early-stage adenocarcinoma and benign conditions including adenoma, but the diagnostic accuracy of endoscopic biopsy for SNADETs is low. Furthermore, because the duodenal wall is thin, the biopsy procedure itself can cause unexpected fibrosis and make subsequent endoscopic resection difficult [4, 5]. Therefore, if SNADETs are detected, endoscopic total resection is recommended to obtain conclusive histological diagnosis and complete cure. However, endoscopic resection for SNADETs has not yet been established. Conventional endoscopic mucosal resection (EMR) is the standard method, but its recurrence rate is high at 5–37% [6–10]. Piecemeal EMR is performed for broad lesions but it has a high risk of perforation [11]. Conversely, endoscopic submucosal dissection (ESD) can achieve complete resection regardless of lesion size, but it is also accompanied by high rates of adverse events [12–18, 19••, 20, 21]. Problematic major adverse events are perforation and bleeding. Many countermeasures including new devices and methods have been applied, and the incidence of adverse events is gradually decreasing.

## Clinical features of SNADETs

SNADETs are defined as lesions that are limited to the mucosa or submucosa, and include adenoma and adenocarcinoma. In previous epidemiological surveys, SNADETs were rare [22], and primary duodenal cancers accounted for only about 0.5% of all gastrointestinal malignancies [23]. SNADETs are reported to be detected in less than 0.4% of patients who undergo EGD [24, 25], but recently, they have been detected more frequently because of advances in endoscopy [1•]. Advanced duodenal cancer has a poor prognosis, and surgical treatment is associated with a high rate of morbidity and mortality [26–28]. Goda et al. [1•] reported that the rate of lymph node metastasis in early duodenal cancers was 0% in intramucosal cancers and 10% in submucosal cancers. Intramucosal SNADETs including mucosal cancer and adenoma are a good indication for endoscopic resection, whereas submucosal cancer is an indication for surgical resection with lymph node dissection. Therefore, early diagnosis and treatment of SNADETs are important. Typical endoscopic findings of duodenal adenoma with high-grade atypia or early cancer are tumor diameter larger than 5 mm and red color [1•]. Furthermore, macroscopic types 0–I (protruded type) or 0–IIa + IIc (flat elevated type with shallow depression) with red color are reported as endoscopic features of duodenal submucosal cancer [1•]. These endoscopic diagnostic criteria are expected to increase the EGD detection rates of SNADETs at the stage of adenoma with high-grade atypia or early cancer. This may allow us to improve the poor prognosis of duodenal cancer as well as quality of life, because early detection by EGD promotes less invasive and radical treatment such as endoscopic resection.

## EMR using the snare

At present, conventional EMR using a snare is considered a standard endoscopic therapy for SNADETs, although there are some problems [29]. Reported en bloc resection rate and R0 resection rate of EMR using the snare for SNADETs range from 79 to 98% and from 53 to 62%, respectively [6–10, 19••]. In reported long-term outcome, recurrence rate ranges from 5 to 37% [6–10, 19••]. Intra-EMR perforation rate, delayed perforation rate, and delayed bleeding rate are 0–2%, 0–4%, and 0–15%, respectively [6–11, 19••]. Recently, safer resection methods such as cold snare polypectomy (CSP) [30] and underwater EMR (UWEMR) have been developed [31]. CSP is an easy technique for small duodenal lesions and is expected to reduce the risk of delayed perforation or bleeding because there is no electrothermal damage induced by the high-frequency current. Reported R0 resection rate and perforation rate for small duodenal adenoma less than 6 mm (25 patients) were 68% and 0%, respectively [30]. UWEMR developed by Binmoeller et al. [31] is a unique technique that resects the target lesions underwater by snare without lifting them with submucosal injections. Because the gastrointestinal tract is not stretched by air inflation, it is expected that capturing a target lesion of up to 2 cm in water is easier in the duodenum. Reported R0 resection rate and perforation rate of small duodenal adenoma smaller than 20 mm (30 patients) were 61% and 0%, respectively [32]. A large Japanese multicenter retrospective study (1397 patients) showed that R0 resection rates and perforation rates of EMR, CSP + UWEMR, ESD, and laparoscopic-endoscopic cooperative surgery (LECS) were 53% and 2.3%, 44% and 3.4%, 75% and 16.2%, and 74% and 7.7%, respectively [19••]. At present, EMR using a snare, including CSP and UWEMR, is recognized as an easy and safe technique for small SNADETs. However, since EMR is a resection method performed by narrowing down the snare, it is difficult to control the excision line and it frequently leads to piecemeal EMR. If there is recurrence after piecemeal EMR, because of severe fibrosis caused by EMR, excision is difficult, even with ESD, and the risk of perforation is high. Incomplete excision with EMR can create a situation that requires invasive surgery including pancreaticoduodenectomy. Theoretically, EMR is considered an alternative to ESD until a system that can safely enforce ESD is established.

## ESD

**Fig. 1:**
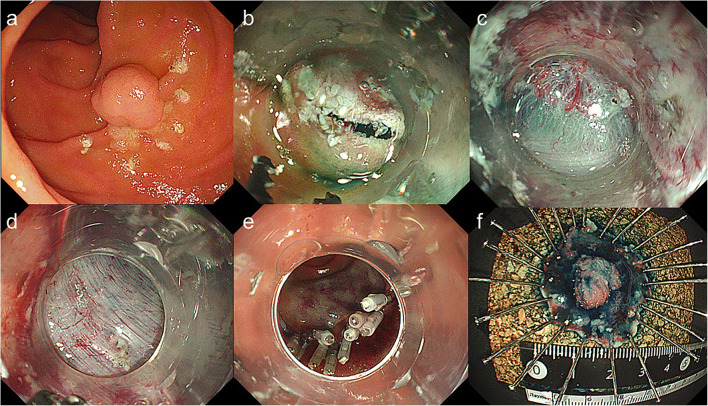
Endoscopic submucosal dissection (ESD) using the short-type Clutch Cutter. **a** Endoscopic view of a 0–I + IIa–type tumor located in the descending duodenum. Marks are made at several points along the outline of the lesion with a forced coagulation current. **b** Endoscopic view of the mucosal incision using the short-type Clutch Cutter after submucosal injection. **c** Endoscopic view of the submucosal dissection using the short-type Clutch Cutter. The submucosal tissue being grasped, pulled, and excised from the muscle layer. **d** The lesion is removed completely from the muscle layer. **e** The post-ESD ulcer that has been completely closed with endoscopic clips. **f** The resected specimen shows en bloc resection of the lesion.

Compared with EMR for early gastrointestinal tract cancer, ESD has considerable advantages regarding the en bloc resection rate, R0 resection rate, and rate of local recurrence [33–38]. EMR has gradually been replaced by ESD. EMR resects the lesion by narrowing down the oval snare; therefore, it is difficult to seize the tumor completely according to the shape and size of the lesion, and the en bloc resection rate is low [33]. In contrast, ESD resects targeted tissue little by little using a knife or scissors (Video 1) while observing the demarcation line of the lesion with an endoscope (Fig. 1); thus, ESD provides high R0 resection rates and low local recurrence rate, irrespective of tumor size and shape. ESD has become a standard treatment for superficial esophageal, gastric, and colorectal tumors in Japan. Reported en bloc resection rate and R0 resection rate of ESD for SNADETs range from 67 to 100% and from 29 to 90%, respectively (Table 1) [8, 12–18, 19••, 20, 21]. In reported long-term outcome, the recurrence rate of ESD for SNADETs was 0% [8, 13–15, 17, 18, 20]. The en bloc resection rate of ESD is higher than that of EMR, which contributes to accurate histological evaluation of the resected specimen, and high R0 resection rate and low local recurrence rate [8, 15, 39]. However, ESD for SNADETs is technically demanding because of organ specificity of the duodenum and it is reported to have a high incidence of severe, including fatal, adverse events [40]. At present, SNADET is a rare disease, and ESD techniques for duodenal tumors have not yet been established and fully evaluated for their effectiveness and safety. The above clinical outcomes for duodenal ESD were mainly reported by skilled endoscopists at high-volume centers in Asia including Japan [12–18, 19••, 20, 21].

**Table 1:** Reported clinical outcome of endoscopic submucosal dissection using various devices for superficial non-ampullary duodenal epithelial tumors

Author	Year	Main device	Number of patients	Median size mm	En bloc (R0) resection rate	Perforation rate (*n*)	Delayed bleeding rate (*n*)	Surgical* intervention rate (*n*)	Recurrence rate (*n*)
Honda et al. [12]	2009	Hook knife	9	22	100% (ND)	22% (2)	33% (3)	11% (1)	ND
Jung et al. [13]	2013	Insulated tip knife	14	10	79% (ND)	36% (5)	0% (0)	14% (2)	0% (0)
Hoteya et al. [14]	2013	Dual knife	41	26	ND (90%)	39% (16)	18% (7)	10% (4)	0% (0)
Nonaka et al. [15]	2014	Insulated tip knife	8	12	86% (57%)	25% (2)	0% (0)	13% (1)	0% (0)
Yamamoto et al. [8]	2014	Insulated tip knife	30	14(mean)	100% (90%)	10% (3)	0% (0)	3% (1)	0% (0)
Minoda et al. [16]	2015	Clutch Cutter	7	20	100% (29%)	0% (0)	0% (0)	0% (0)	ND
Ishii et al. [17]	2015	Flex knife/Hook knife	16	13	94% (81%)	6% (1)	0% (0)	6% (1)	0% (0)
Park et al. [18]	2015	ND	6	8	67% (ND)	33% (2)	0% (0)	0% (0)	0% (0)
Ono et al. [19••]	2016	ND	445	ND	91% (75%)	16% (72)	3% (24)	5% (24)	ND
Miura et al. [20]	2017	Hook knife	28	23	100% (86%)	7% (2)	4% (1)	0% (0)	0% (0)
Dohi et al. [21]	2019	Clutch Cutter	47	15	100% (98%)	2% (1)	4% (2)	0% (0)	ND

*ND* not described

*Surgical intervention due to ESD related complications

## Adverse events of ESD for SNADETs

ESD is technically more difficult for duodenal tumors than tumors of other organs, with a higher rate of severe adverse events. The difficulty of duodenal ESD is caused by anatomical and histological features of the duodenum. Anatomical problems are redundant stomach and duodenal angle, which cause poor control of the endoscope and a vertical approach to the muscularis propria during ESD. Histological problems are the presence of Brunner’s glands and thin muscularis propria that lead to poor submucosal elevation after submucosal injection and easy full-thickness electric damage of the muscle layer [4, 5, 40]. Reported intraoperative and delayed perforation rates are 19–35% and 3–20%, respectively. Incidence of delayed bleeding is reported as 0–22%. [13, 14, 19••, 41–43]. The major cause of intraoperative perforation is unintentional electrothermal damage to the thin muscularis propria, probably caused by electromechanical problems of the ESD devices, coupled with poor endoscopic control and intraoperative endoscopic view. The main causes of delayed perforation and bleeding are speculated to be excessive electrocauterization of the duodenal wall caused by electromechanical problems of the ESD devices, and exposure of the ulcer base to pancreatic and bile juices, which may cause proteolysis or chemical irritation [4, 5, 20, 40].

## Intraoperative countermeasures for adverse events

### Selection of ESD devices from the point of view of electric mechanism

**Fig. 2:**
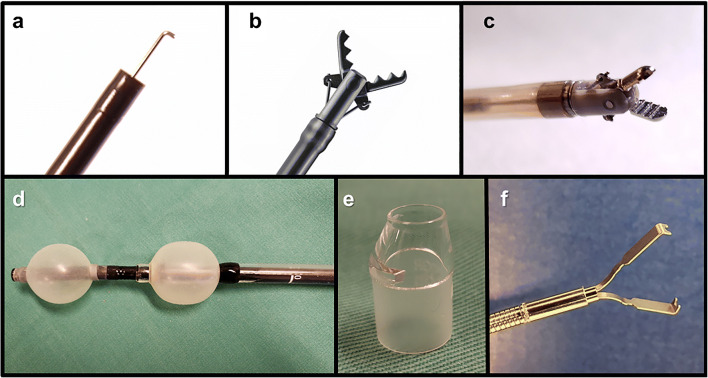
Preventive endoscopic devices for adverse events associated with duodenal endoscopic submucosal dissection. **a** Hook knife. **b** Short-type Clutch Cutter (grasping-type scissors forceps). **c** Tighturn (bipolar hemostatic forceps). **d** Short-type double-balloon endoscope. **e** ST hood (small-caliber-tip transparent hood). **f** ZEOCLIP (ZP-CL; ZEON MEDICAL, Tokyo, Japan) (single opening-and-closing clips).

In duodenal ESD, the approach to go straight to the muscular layer is frequently taken, so the use of a conventional knife that incises while pressing lightly on the muscle layer increases the risk of perforation. Therefore, it is considered safe to perform ESD using a hook knife [17] (Fig. 2a) (KD 620LR; Olympus, Tokyo, Japan) or a Clutch Cutter (grasping-type scissors forceps) [16, 38] (Fig. 2b and Video 1) (DP2618DT-35; Fujifilm, Tokyo, Japan) that can be energized and incised while pulling after hooking or grasping tissue. Since these two devices can energize the targeted tissue away from the thin muscle layer, there is a low risk of electrothermal damage to the muscle layer. Reported perforation rates of hook knife [12, 20] and Clutch Cutter [16, 21] were 7–22% and 0–4%, respectively (Table 1). Furthermore, hemostatic coagulation for intra-ESD bleeding is another main cause of postoperative perforation. Conventional hemostatic forceps use a monopolar electrical current system and have no outside insulation. Although they have a strong coagulation effect for broad and deep tissue, their use leads to postoperative perforation. For duodenal ESD, it is desirable to use safe outer-side insulated grasping-type scissors forceps (Clutch Cutter) [16, 21] (Fig. 2b) or bipolar hemostatic forceps [44] (Fig. 2c) (Tighturn, RH7C2900; ZEON MEDICAL, Tokyo, Japan), which allow pinpoint coagulation of the bleeding artery or vein.

### Endoscopic approach method from the point of view of better endoscopic control

Hayashi et al. reported the unique technique “the pocket creation method” [45] which provides us better scope control during ESD. In this method, a 2–3-cm mucosal incision is made on the endoscopic side of the lesion. This is followed by submucosal dissection of the same part to create a cavity (pocket) where there is sufficient space available for sufficient insertion and fixation of the endoscope. In the pocket space, the endoscope during ESD is stabilized in the submucosa and the endoscopic view allows for a horizontal approach to the muscularis propria layer. Miura et al. [20] reported 100% en bloc resection rate and 4% perforation rate of duodenal ESD using this method.

Another unique approach is the use of a double-balloon endoscope. Originally, the double-balloon endoscope was developed by Yamamoto for small intestinal endoscopy [46]. The short-type double-balloon endoscopes (Fig. 2d) (EI-580BT; Fujifilm) can keep the digestive tract short. Thus, the use of double-balloon endoscopes improves anatomical problems such as redundant stomach and duodenal angles that cause poor endoscopic control and a vertical approach to the muscularis propria layer [40]. This is a promising way to achieve accurate and safe ESD of the duodenum by stabilizing the control of the endoscope tip.

### Endoscopic observation method from the point of view of better intraoperative visualization

The presence of Brunner’s glands means that sufficient thickness of the submucosa cannot be obtained even after submucosal injection, so it is difficult to insert the tip of the endoscope into the submucosa after mucosal incision by ESD using a conventional hood. Therefore, submucosal excision is performed under poor endoscopic observation and this situation leads to perforation. To overcome this, the small-caliber-tip transparent (ST) hood (Fig. 2e) (Video 1) (DH-40GR; Fujifilm) is important. Since the tip of the ST hood is tapered, it can easily be inserted into the submucosa from a narrow mucosal incision, and submucosal dissection can be performed under direct endoscopic visualization. It provides adequate countertraction to the submucosal layer and good visualization of the targeted submucosa during ESD [20, 40].

One more unique technique is the water pressure method [47]. In this procedure, the waterjet function of the endoscope is used, and the water pressure helps insertion of the tip of the hood and provides clear vision underwater during ESD.

## Postoperative countermeasures for adverse events

### Endoscopic closure of the post-ESD ulcer using clips

Closure of the duodenal superficial tissue defect after ESD (Fig. 1e) is considered to be the most effective countermeasure for post-ESD perforation and delayed bleeding, because it protects against exposure of electrically damaged muscle layers to pancreatic and bile juices. Kato et al. [48] reported that a complete closure of the mucosal defect after duodenal ESD significantly decreased the number of delayed adverse events as compared with the incomplete/no closure group. Reported rates of post-ESD perforation in the complete closure and incomplete/no closure groups were 1.7% and 10.5%, respectively. Reported rates of delayed bleeding in each group were 0% and 10.5%, respectively. Another study showed that simple prophylactic closure using an endoclip after duodenal ESD reduces the risk of delayed bleeding [49]. Fukuhara et al. [50] reviewed 32 patients with intraoperative perforation of duodenal ESD. Those who could be completely closed had significantly shorter fasting and hospitalization periods and significantly lower maximum serum C-reactive protein levels than those who could not. At present, the closure of mucosal defects after duodenal ESD using endoclips (Fig. 2f) is recognized as the most effective countermeasure for perforation and delayed bleeding of duodenal ESD.

### Other methods

#### Over-the-scope clip

In addition to being a hemostatic device for bleeding lesions, the over-the-scope clip (OTSC) (Ovesco Endoscopy AG, Tübingen, Germany) was developed for closure of mucosal defects in patients with acute gastrointestinal perforation and anatomical leakage [51] and is used to close duodenal mucosal defects after ESD [52]. Tashima et al. [53] investigated the clinical outcome of 50 patients with prophylactic closure using OTSC after duodenal ESD. They reported a closure success rate of 94%, delayed perforation rate of 2.1%, and delayed bleeding rate of 6.3%.

#### Endoscopic shielding method with polyglycolic acid sheets and fibrin glue

Polyglycolic acid (PGA) sheets and fibrin glue are commonly used to cover open wound surfaces in the surgical field [54]. Furthermore, combination of PGA sheets (Neoveil; Gunze, Osaka, Japan) and fibrin glue (Beriplast P Combi-Set; CSL Behring Pharma, Tokyo, Japan) has proven effective in prevention of various adverse events after ESD in the esophagus, stomach, and colorectum [55–57]. Similarly, some case reports have shown the efficacy of shielding over ulcers after duodenal ESD [58–60]. However, a Japanese prospective multicenter randomized controlled trial (137 patients) [61] showed that the PGA shielding method did not significantly prevent post-ESD bleeding. Post-ESD bleeding occurred in three (4.5% patients) in the PGA group and four (5.7%) in the control group. There was no significant difference between the two groups. At present, this procedure may be considered when endoscopic closure using the clips is unsuccessful.

#### LECS

LECS was developed as a less invasive surgical procedure for gastric submucosal tumors [62]. LECS is also applied to early-stage duodenal tumors to reduce the risk of complications. In LECS for SNADETs [63], the mucosal defect after ESD is closed tightly after laparoscopic suturing of the duodenal wall from the extraluminal side. No severe adverse events have been reported for this procedure. If intra-ESD perforation occurs, it can be sutured endoscopically and laparoscopically. However, this technique needs many specialists including endoscopists, anesthesiologists, and surgeons, and costs too much. Furthermore, LECS cannot cope with lesions on the inner side of the duodenum with the pancreatic head parenchyma behind [64, 65].

#### Endoscopic nasobiliary and pancreatic duct drainage

Pancreatic and bile juices are an important cause of delayed perforation [4, 40, 48]. Theoretically, external drainage of bile and pancreatic juices might be effective in preventing delayed perforation. The preventive efficacy of endoscopic nasobiliary and pancreatic duct drainage (ENBPD) for ulcers after duodenal ESD has only been shown in a small number of case series [50]. Furthermore, ENBPD has a risk of post-ESD ulcer perforation resulting from insertion of a side-viewing endoscope, as well as post-endoscopic retrograde cholangiopancreatography pancreatitis. The indications for ENBPD should be limited to cases in which endoscopic closure using clips is unsuccessful or impossible.

## Conclusion

ESD is the only endoscopic treatment that can reliably perform R0 resection of mucosal cancer and precancerous lesions of the entire gastrointestinal tract regardless of their size or shape. At present, ESD for SNADETs is a challenge, because it has the difficulty of ESD procedures and the occurrence of serious adverse events caused by the anatomical and histological characteristics of the duodenum. However, the R0 resection rate and safety have improved because of the development of new devices and techniques. Prospective studies are necessary to determine if ESD can become a part of the standard of care for SNADETs.

## Electronic Supplementary Material

Video 1 VTR of the procedure of endoscopic submucosal dissection using the short type Clutch Cutter for broad flat type intramucosal duodenal cancer of the bulbus. (WMV 231167 kb)
